# Editorial: Gene editing in cancer gene therapy

**DOI:** 10.3389/fmed.2026.1797737

**Published:** 2026-03-04

**Authors:** Yuyue Hou, Yan Zhou, Keshan Wang, Xiaotian Xia

**Affiliations:** 1 Department of Nuclear Medicine, Union Hospital, Tongji Medical College, Huazhong University of Science and Technology, Wuhan, Hubei, China; 2 Hubei Province Key Laboratory of Molecular Imaging, Wuhan, Hubei, China; 3 Key Laboratory of Biological Targeted Therapy, The Ministry of Education, Wuhan, Hubei, China; 4 Department of Urology, Union Hospital, Tongji Medical College, Huazhong University of Science and Technology, Wuhan, China

**Keywords:** cancer gene therapy, CAR-T cell therapy, CRISPR-Cas9-based tool, delivery systems, gene editing

Gene editing technologies have rapidly evolved from experimental tools into promising therapeutic strategies in oncology. Advances in programmable nucleases, epigenome-targeting approaches, and delivery systems are reshaping how cancer is studied and treated. This *Research Topic, Gene Editing in Cancer Gene Therapy*, brings together five complementary contributions—four *Review articles* and one *Original Research article*—that collectively reflect the current momentum, challenges, and translational trajectory of gene-based interventions in oncology.

The five articles included in this *Research Topic* reflect the breadth and growing maturity of the field. Rather than concentrating on a single editing platform or cancer type, the contributions collectively illustrate how gene editing strategies are being integrated into broader oncologic frameworks, spanning genome modification, epigenetic regulation, and combination therapies.

At the mechanistic and conceptual foundation of the Topic, Zhang et al. provide a comprehensive review of epigenetic regulation and epigenetic gene editing tools. Starting from fundamental principles of epigenetic inheritance and core regulatory mechanisms, the authors trace the evolution from traditional gene editing toward epigenetic editors, including KRAB- and DNMT-based systems. Importantly, this review goes beyond tool description by critically analyzing key bottlenecks such as editing efficiency, specificity, long-term safety, and delivery constraints. By discussing both neoplastic and non-neoplastic disease contexts, the article highlights epigenetic editing as a versatile and potentially safer alternative for multigene and regulatory disorders, with clear implications for cancer therapy (Zhang et al.).

Expanding the scope to broader therapeutic strategies, Youssef et al. review the evolving role of gene therapy in oncology, with a strong emphasis on immune modulation. This article synthesizes advances across multiple platforms, including gene replacement, gene silencing, oncolytic virotherapy, CAR-T cell therapy, and CRISPR-Cas9-based editing. Particular attention is given to how genetic interventions can enhance antitumor immunity, overcome immune evasion, and address tumor heterogeneity. The review also discusses emerging concepts such as ferroptosis induction and combination strategies involving immune checkpoint inhibitors. In addition to scientific advances, the authors examine regulatory frameworks and ethical considerations, underscoring the importance of governance and accessibility as gene therapies move toward broader clinical adoption.

From a delivery and materials science perspective, Wang et al. focus on nanogene editing drug delivery systems, using liver fibrosis as a disease model. Although centered on fibrotic disease, this review addresses challenges highly relevant to oncology, including targeted delivery, expression efficiency, and safety of gene editing payloads. By summarizing commonly used gene editing targets and nano-delivery formulations, the article highlights how advances in delivery platforms may enable more precise and effective genetic interventions in complex tissue environments, including solid tumors (Wang et al.).

The *Research Topic* also incorporates a policy and practice-oriented contribution by Delisle et al., which examines the development of cell therapy, gene therapy, and regenerative medicine in Québec, Canada. Drawing on insights from a multidisciplinary symposium, this article synthesizes perspectives from scientists, industry stakeholders, ethicists, and regulators. The authors identify strategic priorities spanning infrastructure, workforce training, regulatory adaptation, reimbursement frameworks, and equitable access (Delisle et al.). This contribution emphasizes that successful translation of gene editing technologies depends not only on scientific innovation but also on coordinated health system and policy support.

Anchoring these reviews in clinical genomics, Kawanaka et al. present the sole Original Research article in the Topic, investigating the prognostic impact of homologous recombination DNA repair (HRR) gene alterations in pancreatic ductal adenocarcinoma. Through large-scale genomic profiling, the authors demonstrate that HRR-altered tumors exhibit distinct mutation patterns and improved overall survival, while coexisting driver mutations carry differential prognostic significance. This study illustrates how genomic stratification can inform personalized treatment strategies and provides a clinical rationale for future gene-targeted and genome-guided interventions (Kawanaka et al.).

Collectively, these five contributions portray gene editing in cancer therapy as an integrated continuum, from epigenetic regulation and immune modulation to delivery technologies, clinical genomics, and policy frameworks. Rather than focusing on a single technology, this *Research Topic* highlights the inherently interdisciplinary nature of gene editing and its expanding role in precision oncology.

We hope that this *Research Topic* will serve as a valuable resource for researchers and clinicians seeking to understand the evolving landscape of gene editing in oncology, and that it will stimulate further interdisciplinary efforts bridging molecular engineering, cancer biology, and translational medicine.

